# Impact of Close-Kin Experience with Metabolic Bariatric Surgery on Patient Outcomes: A Retrospective Cohort Study from the Middle East

**DOI:** 10.1007/s11695-025-08073-3

**Published:** 2025-08-15

**Authors:** Gabriela Restrepo-Rodas, Juan S. Barajas-Gamboa, Maryam Al Zubaidi, Nada Mahmoud, Mohammed Abdallah, Alfredo D. Guerron, Javed Raza, Juan Pablo Pantoja, Carlos Abril, Ricard Corcelles, Gabriel Diaz Del Gobbo, Matthew D. Kroh, John Rodriguez

**Affiliations:** 1 Cleveland Clinic Abu Dhabi, Abu Dhabi, United Arab Emirates; 2 College of Medicine and Health Sciences, Khalifa University of Science and Technology, Abu Dhabi, United Arab Emirates; 3 Case Western Reserve University, Cleveland, OH USA; 4 Cleveland Clinic, Cleveland, OH USA

**Keywords:** Close-kin patients, Bariatric surgery, Social history

## Abstract

**Background:**

Metabolic bariatric surgery (MBS) is increasingly common worldwide and in the Middle East due to its safety, efficacy, and durability. While close-kin patient (CKP) referrals from relative who have undergone MBS are frequent, the impact of this relationship remains unclear. This study evaluates whether CKP referrals influence BS outcomes and follow-up compliance at a tertiary medical center in the UAE.

**Methods:**

A population-based study was conducted with IRB approval. Primary BS cases from January 2017 to December 2018 were included. Patients were contacted by phone to collect CKP information and classified into those with close kin relatives who underwent BS (YCK) and those without (NCK).

**Results:**

Of 235 patients, 121 agreed to participate. The YCK group included 82 (67.7%) patients, mostly female (73%), mean age 39 ± 13 years, and initial BMI 44.6 ± 7.47 kg/m². The NCK group included 39 (32.2%) patients, mostly female (79%), mean age 38 ± 14 years, and initial BMI 46.9 ± 6.73 kg/m². Patients in the YCK group were less likely to experience late complications (p 0.05). Follow-up at one year was significantly higher in the YCK group (82.93% vs. 62%, p 0.05). Among YCK patients, 87.8% reported their relative had a positive experience (PYCK), and 12.1% reported a negative experience (NYCK). There was no significant correlation between relatives’ experiences and patients’ outcomes.

**Conclusion:**

Patients with close kin referrals have similar early outcomes but show better postoperative care adherence, which may contribute to reduced late complication risks; though further studies are needed to confirm this finding.

## Introduction

Obesity has emerged as a critical global health crisis. It affects millions of individuals and families worldwide causing an epidemic that brings substantial physical, psychological, and socioeconomic burdens. In the United Arab Emirates (UAE), the prevalence of obesity and overweight among adults is alarmingly high, estimated at 27.8% and 40.1% respectively, with a higher prevalence observed among females [1]. Metabolic bariatric surgery (MBS) has been established as the most effective long-term treatment for severe obesity and its associated comorbidities [2]. Over the last decade, MBS has gained significant popularity in the UAE and the broader Middle East region, making it necessary to understand the factors influencing its outcomes in this specific context [3, 4].

Various factors, including personal experiences and social connections, often influence the decision to undergo MBS. Pratt et al. introduced the concept of the"Social History of Metabolic Surgery"to describe patients who initiate the metabolic surgery pathway based on the experiences of close kin relatives who have undergone BS [5]. They estimated that a striking 83% to 85% of patients fall into this category. While these findings are significant, they were primarily based on data from Western populations, where individual decision-making tends to dominate healthcare choices. In contrast, in the Middle East, particularly within Arab cultures, the influence of close kin on medical decisions may be more pronounced due to the collective nature of these societies [6, 7]. Family members often play a central role in guiding healthcare decisions, which can profoundly affect both the decision to undergo metabolic bariatric surgery and the patient's adherence to post-operative care [8].

The success of MBS relies on more than the procedure; post-operative follow-up is vital for monitoring progress, managing complications, and ensuring adherence to treatment [9]. In Middle Eastern cultures, where family support is key, involving relatives who have undergone this type of surgery may improve follow-up adherence and enhance long-term outcomes [10–12]. This suggests that the family’s role in healthcare may influence not only the decision to undergo surgery but also the patient’s long-term commitment to post-operative care, thereby potentially enhancing the overall success of bariatric interventions in this population.

Despite the apparent importance of close-kin experiences with metabolic bariatric decision-making, there is still limited evidence published in the medical literature on how these experiences influence the pre- and postoperative course of bariatric patients. Therefore, understanding the impact of close-kin experience on metabolic bariatric surgery outcomes is particularly relevant for current clinical practice. This study aims to evaluate whether close-kin patient (CKP) experience with MBS influences the outcomes and follow-up adherence of patients at a tertiary referral academic medical center in the United Arab Emirates.

## Methods

### Study Design and Ethical Approvals

This study is a retrospective review of a prospective registry. Data were collected for patients who underwent metabolic and surgery between January 2017 and December 2018. Electronic medical records were reviewed to gather data on demographic characteristics, preoperative obesity-related conditions, postoperative outcomes, and follow-up adherence. Additionally, prospective data collection was performed via phone call interviews to evaluate the close-kin patient (CKP) relationship and its correlation with surgical outcomes. This study was approved by our institution's Research Ethics Committee (REC) under the internal number A-2017–029.

### Objectives

The primary objective of this study was to evaluate whether close-kin patient (CKP) experience influences MBS outcomes, including complication rates, BMI changes, and one-year weight loss outcomes, in patients with (Yes Close Kin group—YCK) and without (No Close Kin group—NCK) close-kin history of MBS at a tertiary referral academic medical center in the United Arab Emirates. Secondary objectives included comparing baseline characteristics such as age, gender, initial BMI, and type of bariatric surgery between groups to explore potential differences. Multivariate logistic regression analyses were performed to identify predictors of early and late postoperative complications.

### Definitions

- Close-Kin: First-degree relatives (parents, siblings, children) and second-degree relatives (grandparents, aunts, uncles, cousins) who had undergone MBS.
- Positive Experience: Characterized by substantial weight reduction with no major complications or minor complications that did not adversely affect postoperative recovery.
- Negative Experience: Cases with insufficient weight reduction or occurrence of complications affecting quality of life.
- Early complications: those occurring within 30 days postoperatively, including ER visits and reoperations.
- Late complications: Those occurring beyond the 30-day postoperative period.

### Population and Inclusion/Exclusion Criteria

Patients who underwent MBS during the study period were contacted to determine their status as CKPs. Participants were categorized into two cohorts based on their family history of metabolic bariatric surgery. The YCK group consisted of patients who had at least one close-kin relative, such as a parent, sibling, child, grandparent, aunt, uncle, or cousin, who had previously undergone MBS. In contrast, the NCK group included patients with no such family history. The study included all patients who underwent primary metabolic bariatric surgery during the designated timeframe, irrespective of age. Exclusion criteria were applied to patients undergoing revision bariatric procedures and those who declined to participate when contacted for follow-up.

### Telephone Interview Protocol

Trained research assistants conducted standardized telephone interviews to explore participants’ relationships with their CKPs who had undergone MBS. Participants were asked about their perception of their relatives’ surgical outcomes—categorized as either positive or negative—and how these outcomes influenced their decision to pursue metabolic bariatric surgery. Responses were recorded using a standardized form and subsequently categorized for analysis.

### Data Collection

The study employed a combination of retrospective and prospective data collection methods. Retrospective data included patient demographics, obesity-related conditions, operative times, length of hospitalization, reoperations, complications, weight loss profiles, and follow-up visits. Prospective data were collected through structured telephone interviews to gather information on CKPs and patients’ experiences.

### Statistical Analysis

Descriptive statistics were computed for all variables. Frequencies and percentages were calculated for categorical variables, while mean, and standard deviation were calculated for quantitative variables. Comparisons were completed using univariate parametric or non-parametric methods where appropriate, with a significance level of *p* < 0.05 to determine statistical significance. Independent sample t-tests were used to examine continuous variables, and Fisher's exact test was used for dichotomous variables. Multivariable logistic regression models were used to evaluate potential predictors of early and late complications. All analyses were carried out using R (version 2.13 or higher, The R Foundation for Statistical Computing, Vienna, Austria).

## Results

A total of 235 patients underwent MBS from January 2017 to December 2018, with 121 (51.4%) agreeing to participate in this study (Table 1). The YCK group comprised 82 (67.7%) patients, mostly female (73%), with a mean age of 39 ± 13 years and an initial BMI of 44.6 ± 7.47 kg/m^2^. The NCK group included 39 (32.2%) patients, mostly female (79%), with a mean age of 38 ± 14 years and an initial BMI of 46.9 ± 6.73 kg/m^2^. Dyslipidemia (55%) was the most common obesity-related condition in the YCK group, while obstructive sleep apnea (49%) was most common in the NCK group. The most common procedure in the YCK group was sleeve gastrectomy, while in the NCK group it was roux-en-y gastric bypass. There was no significant difference in procedure length between the groups (126.6 ± 48.65 min vs. 139 ± 51.3 min, *p* = 0.19) or in intraoperative findings.

**Table 1:** Baseline characteristics

Variables	YCK (N = 82)	NCK (N = 39)	*p* Value
Age (years, mean, SD)	39 ± 13	38 ± 14	0.64
Male (N, %)	22 (27%)	8 (21%)	0.50
Female (N, %)	60 (73%)	31 (79%)	0.50
Initial BMI (mean, kg/m2)	44.6 ± 7.47	46.9 ± 6.73	0.10
Initial Weight	115.9 ± 21.9	122.2 ± 25.61	0.16
**Obesity-Related Conditions**			
Hypertension (N, %)	35 (43%)	13 (33%)	0.42
Dyslipidemia (N, %)	45 (55%)	16 (41%)	0.17
Diabetes type 2(N, %)	26 (32%)	17 (44%)	0.22
GERD (N, %)	24 (29%)	10 (26%)	0.82
OSA (N, %)	28 (34%)	19 (49%)	0.16
**Surgical Technique**			
RYGB	34 (41.4%)	22 (56%)	0.17
LSG	48 (58.54%)	17 (44%)	0.17
Procedure Length	126.6 ± 48.65	139 ± 51.3	0.19

*YCK* yes close-kin, *NCK* no close kin, *N* number, *SD* standard deviation, *BMI* body mass index, *GERD* gastroesophageal reflux disease, *OSA* obstructive sleep apnea, *RYGB* Roux-en-Y gastric bypass, *LSG* Laparoscopic sleeve gastrectomy

Analysis of perioperative outcomes revealed no statistically significant differences between the two cohorts (Table 2). The mean length of hospital stay did not differ significantly, with patients from the YCK group staying for an average of 2.6 days compared to 3.9 days for the NCK group (*p* = 0.06). Early minor complications were observed in 3.6% of patients in the YCK group and 8% of patients in the NCK group, with no significant difference between groups (*p* = 0.70). The most common early minor complications in both groups were nausea/vomiting and urinary tract infections. Similarly, early major complications occurred in 4.8% of patients in the YCK group and 3% of patients in the NCK group, with no significant difference. The most common early major complication in both groups was gastrointestinal bleeding. Even though late complications were higher in the NCK group (10%) than in the YCK group (3.66%), this difference was not statistically significant (*p* = 0.21). Follow-up adherence was significantly higher at one year follow up in patients from the YCK group (82.9%) than in the NCK group (62%, *p* = 0.009).

**Table 2:** Comparison postoperative outcomes

Variables	YCK (N = 82)	NCK (N = 39)	*p* Value
**Length of stay**	**2.6 ± 1.2**	**3.9 ± 5.8**	**0.06**
**Early minor complications**	**3 (3.6%)**	**3 (8%)**	**0.70**
Nausea and vomiting	2 (2.4%)	0 (0%)	0.32
Urinary tract infection	1 (1.2%)	2 (5%)	0.19
Trocar/Surgical site infection	0 (0%)	1 (2.5%)	0.14
**Early major complications**	**4 (4.8%)**	**1 (2.5%)**	**0.95**
Gastrointestinal bleeding	4 (4.8%)	1 (2.5%)	0.95
**ED visit in 30 days**	**20 (24.3%)**	**11 (28%)**	**0.66**
**Early Readmission**	**4 (4.8%)**	**1 (2.5%)**	**0.54**
**Early Reoperation**	**4 (4.8%)**	**1 (2.5%)**	**0.54**
**Late complications**	**3 (3.6%)**	**4 (10%)**	**0.14**
Anastomotic stricture	2 (2.4%)	2 (5%)	0.44
Vitamin or mineral deficiency	1 (1.22%)	1 (2.5%)	0.58
Chronic nausea	0 (0%)	1 (2.5%)	0.14
**Follow up at 1 year**	**68 (82.9%)**	**24 (62%)**	**0.00**
**Follow up at two years**	**7 (8.5%)**	**7 (18%)**	**0.13**
**Follow up more than two years**	**6 (7.3%)**	**6 (15%)**	**0.16**

*YCK* yes close kin, *NCK* no close kin, *ED* emergency department

Sixty-eight patients of the YCK group and thirty-four patients from the NCK group, returned for the one-year follow-up. (Table 3). The YCK group had a weight of 86.4 ± 16.9 kg, while the NCK group had a weight of 83.3 ± 17.84 kg (*p* = 0.45). Patients in both groups had a similar BMI (33 ± 6.64 vs. 32.6 ± 5.21 kg/m^2^ (*p* = 0.77). However, the change in BMI was statistically different, with patients in the YCK group having a reduction of 12.1 ± 3.82 points in comparison with the NCK group which had a change of 14 ± 4.8 (*p* = 0.03). The difference of total weight loss and excess weight loss was not different among the two groups (*p* = 0.08 and *p* = 0.59, respectively).

**Table 3:** Weight loss profile at 12 months

Variables	YCK (N = 68)	NCK (N = 34)	*p* Value
Weight at 12 months	86.1 ± 17.3	83.3 ± 17.8	0.45
BMI at 12 months	33 ± 6.6	32.6 ± 5.2	0.77
Change in BMI	12.1 ± 3.8	14 ± 4.8	0.03
%TBWL	26.9 ± 7.3	29.7 ± 8.3	0.08
%Excess weight loss	52.5 ± 16.7	54.3 ± 15.8	0.59

*YCK* yes close kin, *NCK* no close kin, *BMI* body mass index, *TBWL* total body weight loss

### Multivariable Logistic Regression

A multivariable logistic regression was performed to evaluate independently predictive variables of early complications and late complications. For early complications, the only predictive variable was gender, with females being less likely to experience early complications than males (OR 0.19, 95%CI 0.04–0.9, *p* = 0.03, Table 4). For late complications, three variables were independently predictive of late complications (Table 5). Patients in the YCK group had significantly lower odds of experiencing complications, as well as those with diabetes and who underwent RYGB.

**Table 4:** Early complications on multivariable logistic regression

Variable	Odds Ratio	95% Confidence Interval	p-value
Group (YCK, NCK)	0.48	(0.1004, 2.2573)	0.35
Age	1.03	(0.9622, 1.1013)	0.39
Gender	0.20	(0.0429, 0.9004)	0.03
BMI	1.00	(0.9032, 1.1029)	0.97
Hypertension	2.53	(0.4005, 16.0414)	0.32
Dyslipidemia	0.51	(0.1016, 2.5827)	0.41
Diabetes	0.56	(0.0978, 3.2516)	0.52
GERD	1.25	(0.2411, 6.4777)	0.79
OSA	0.98	(0.2175, 4.3796)	0.97
Surgery	0.41	(0.0835, 2.0362)	0.27

*YCK* yes close kin, *NCK* no close kin, *BMI* body mass index, *GERD* gastroesophageal reflux disease, *OSA* obstructive sleep apnea

**Table 5:** Late complications on multivariable logistic regression

Variable	Odds Ratio	95% Confidence Interval	*p*-value
Group (YCK, NCK)	0.0914	(0.0086, 0.9658)	0.04
Age	1.1099	(0.9956, 1.2372)	0.06
Gender	0.7918	(0.0749, 8.3652)	0.84
BMI	1.1136	(0.9617, 1.2895)	0.15
Hypertension	2.0993	(0.1669, 26.4051)	0.56
Dyslipidemia	0.7359	(0.0627, 8.6343)	0.80
Diabetes	0.0215	(0.0006, 0.7572)	0.03
GERD	0.2013	(0.0157, 2.5739)	0.21
OSA	0.3341	(0.0246, 4.5355)	0.41
Surgery	0.0251	(0.0007, 0.8523)	0.04

*YCK* yes closekin, *NCK* no close kin, *BMI* body mass index, *GERD* gastroesophageal reflux disease, *OSA* obstructive sleep apnea

### Subgroup Analysis: Positive Experience vs. Negative Experience

Among the 82 patients with a close-kin relative who underwent BS, 72 (87.8%) reported their relative had a positive experience (Positive Yes Close Kin—PYCK), while 10 (12.1%) reported a negative experience (Negative Yes Close Kin—NYCK) (Table 6). Patients in the PYCK had a lower initial weight than patients in the NYCK group (113.6 ± 19.40 kg vs. 132.3 ± 32.10 kg, *p* = 0.01). Both early and late complications were similar among both groups (*p* = 0.58 and *p* = 0.32, respectively). Twelve months after the surgery, patients in the PYCK group had a weight of 83.9 ± 15.7 kg, compared to the weight achieved by the NYCK group of 97.9 ± 21.73 kg (*p* = 0.01). However, both the change in BMI and the percentage of total weight loss was similar between both groups (*p* = 0.78 and *p* = 0.49, respectively). Similarly, the follow up adherence did not vary between patients with a positive experience and a negative experience. Finally, there was no statistically significant correlation between the experiences of relatives (positive or negative) and the experiences of patients (*p* > 0.05).

**Table 6:** Comparison between positive experience and negative experience

Variables	PYCK (N = 72)	NYCK (N = 10)	p Value
Initial weight	113.6 ± 19.40	132.3 ± 32.10	0.01
Initial BMI	44.1 ± 6.6	48.4 ± 11.5	0.08
Early complications	7 (9.72%)	0 (0%)	0.30
Late complications	2 (2.78%)	1 (10%)	0.25
Weight at 12 months	83.9 ± 15.7	97.9 ± 21.73	0.01
BMI at 12 months	32.4 ± 6.0	36.0 ± 8.9	0.11
Change in BMI	12	12.4	0.77
%TBWL	27.1 ± 7.38	25.4 ± 7.29	0.49
Follow up at 1 year	58 (80.5%)	10 (100%)	0.12
Follow up at two years	21 (29.1%)	1 (10%)	0.20
Follow up more than two years	6 (8.3%)	(0%)	0.34

*PYCK* positive experience in yes close-kin, *NYCK* negative experience in yes close kin, *BMI* body mass index, *TBWL* total body weight loss

## Discussion

This study is among the first to assess the impact of close kin experience with MBS on patient outcomes in the Middle East. While 67.7% of participants had a relative who previously underwent MBS, there were no significant differences in postoperative outcomes or early complications between the YCK and NCK groups. However, the YCK group showed significantly higher follow-up adherence, comparable weight loss profiles and lower late complication rates.

The percentage of close-kin history of MBS in our cohort (67.7%) was lower than the 83–91% reported in other studies [5, 13, 14]. This finding was unexpected, given the collective and family-centered nature of Middle Eastern Arab-Islamic cultures, where extended family ties, typically play a significant role in medical decision-making [10, 11].

Baseline characteristics were comparable between the two groups, with similar initial BMIs and obesity-related conditions. Postoperative outcomes also showed no significant differences in complication rates, consistent with Pratt et al., who found no significant difference in postoperative complications between patients with and without a social history of MBS (*p* = 0.464) appropriate, with a significance level [5]. Notably, follow-up adherence was significantly higher in the YCK group (82.9% vs. 62%, *p* = 0.009), which aligns with previous findings [15]. These findings may be attributed to the social and psychological support provided by relatives who have undergone MBS, highlighting a potential role of familial influence in adherence [8, 12, 16]. However, it is also possible that shared health behaviors, motivations, or healthcare access patterns also contributed to this difference.

The weight loss profile was similar between the groups, except for a greater change in BMI in the NCK group (*p* = 0.03). While potential confounders like small sample size and higher variability in the NCK group should be considered, Slotman’s study supports this finding, showing an improved weight loss profile in patients with a family history of MBS [15].

The logistic regression analysis indicated that gender was identified as a potential predictor in this exploratory analysis, with females having lower odds compared to males (odds ratio = 0.19, *p* = 0.03). This finding is consistent with Husain et al., who identified male gender as an independent predictor of complications after SG or RYGB [17]. This may be related to cultural factors related to Arabic patients, as men in the Middle East might delay seeking medical care or adhere less strictly to postoperative regimens [18]. Given the limited sample size and risk of overfitting, these multivariate models should be interpreted with caution and considered hypothesis-generating rather than confirmatory.

Analysis of predictors of late complications revealed three findings. First, patients in the YCK group appeared less likely to experience late complications compared to the NCK group (*p* = 0.04), potentially due to greater awareness of alarm signs, better adherence to postoperative care, or psychological factors. Though this observation should be interpreted cautiously due to the limited sample size and potential for unmeasured confounding. The literature lacks studies on close kin referrals and late complications, indicating a need for further research. Surprisingly, diabetes was associated with lower odds of complications, possibly due to better management and follow-up, although other confounders should be considered [19]. In contrast to our findings, Hu et al.'s systematic review and meta-analysis found higher late complication rates in RYGB patients compared to LSG, suggesting that our results might be influenced by other confounders, such as higher obesity-related conditions in the LSG group, a small sample size or other factors not fully captured in the study [20].

Comparing positive versus negative experiences within the YCK group revealed that patients in the PYCK group had a lower initial weight than those in the NYCK group (113.6 kg vs. 132.3 kg, *p* = 0.01), possibly due to reluctance to undergo surgery after a relative's negative experience. In fact, Opozda et al. found that observations of others'success or failure were significant factors in deciding on bariatric procedures [21]. Although not statistically significant, it is notable that the NYCK group had a 100% follow-up rate at one year compared to 80.5% in the PYCK group, possibly due to increased awareness of complications in the early postoperative years. It is important to note that these categorizations were based on patients’ subjective reports and were not independently verified. Future studies should aim to use standardized definitions and, where feasible, objective outcome data.

This study is significant as one of the first to examine the impact of close kin referrals on MBS outcomes in the region, which draws a link between social history with postoperative results. The study focused on a culturally unique population providing insights into the influence of familial experiences on patient adherence and late complications. Nonetheless, it has some limitations. Initial non-response bias may have influenced the findings, as not all eligible patients agreed to participate. Similarly, only influence from one close relative per participant was assessed and did not account for the degree of relationship or the presence of multiple kin who underwent surgery. A small sample size may limit the detection of associations and increase the risk of confounding. The observational design limits the ability to establish causality. The reliance on self-reported data for familial history, without an objective evaluation tool, could introduce recall bias. Follow-up attrition may introduce bias and limit generalizability. Moreover, the findings are based on a specific healthcare setting in the UAE and may not be generalizable to other regions with different healthcare systems or cultural dynamics. Future research with larger, more diverse populations and extended follow-up is essential to validate these results. Developing objective tools to evaluate the influence of social history on surgical outcomes could further enhance patient care, leading to more culturally sensitive and effective treatment protocols tailored to the unique social dynamics of Middle Eastern populations.

## Conclusions

Patients with a family history of MBS demonstrated better adherence to postoperative care and appeared to experience fewer late complications, although this finding should be interpreted cautiously. The psychological impact of previous familial experiences also appears to play a role, as patients with relatives who had positive outcomes were more likely to pursue surgery earlier and with better initial weight profiles. These findings highlight the importance of considering familial and social factors when planning and supporting patients through metabolic bariatric surgery, suggesting that leveraging close kin support could enhance patient outcomes and postoperative care adherence.

## Data Availability

No datasets were generated or analysed during the current study.

## References

[1] Ministry of Health & Prevention UAE National Health Survery report 2017–2018.

[2] Sjöström L. Review of the key results from the Swedish Obese Subjects (SOS) trial - a prospective controlled intervention study of surgery. J Intern Med. 2013;273:219–34. 23163728 10.1111/joim.12012

[3] Mezhal F, Oulhaj A, Abdulle A, et al. High prevalence of cardiometabolic risk factors amongst young adults in the United Arab Emirates: the UAE Healthy Future Study. BMC Cardiovasc Disord. 2023;23:137. 36922773 10.1186/s12872-023-03165-3 PMC10015775

[4] Radwan H, Ballout RA, Hasan H, et al. The epidemiology and economic burden of obesity and related cardiometabolic disorders in the United Arab Emirates: a systematic review and qualitative synthesis. J Obes. 2018;2018:1–23. 10.1155/2018/2185942 PMC6311818 30652030

[5] Pratt KJ, Stroup HJ, Breslin L, et al. Social history of bariatric surgery: relationship to patient and associations with postoperative outcomes. Obes Surg. 2023;33:2762–9. 37466828 10.1007/s11695-023-06738-5

[6] Arabiat D, Whitehead L, Al Jabery M, et al. Beliefs about illness and treatment decision modelling during Ill-health in Arabic families. J Multidiscip Healthc. 2021;14:1755–68. 34267524 10.2147/JMDH.S311900 PMC8275164

[7] Alfahmi MZ. Patients’ preference approach to overcome the moral implications of family-centred decisions in Saudi medical settings. BMC Med Ethics. 2022;23:128. 36474278 10.1186/s12910-022-00868-8 PMC9724249

[8] Tymoszuk U, Kumari M, Pucci A, et al. Is pre-operation social connectedness associated with weight loss up to 2 years post bariatric surgery? Obes Surg. 2018;28:3524–30. 30043144 10.1007/s11695-018-3378-6

[9] Wheeler E, Prettyman A, Lenhard MJ, et al. Adherence to outpatient program postoperative appointments after bariatric surgery. Surg Obes Relat Dis. 2008;4:515–20. 18586576 10.1016/j.soard.2008.01.013

[10] Alzubaidi H, Samorinha C, Saidawi W, et al. Preference for shared decision-making among Arabic-speaking people with chronic diseases: a cross-sectional study. BMJ Open. 2022;12:e058084. 35410934 10.1136/bmjopen-2021-058084 PMC9003612

[11] Al-Bahri A, Al-Moundhri M, Al-Mandhari Z, et al. Role of the family in treatment decision-making process for Omani women diagnosed with breast cancer. Patient Educ Couns. 2019;102:352–9. 30170824 10.1016/j.pec.2018.08.026

[12] Kapera O, Xie L, Marroquin E, et al. Association of social support and metabolic bariatric surgery completion among racially and ethnically diverse patients. Obes Surg. 2024;34:2755–63. 38918268 10.1007/s11695-024-07343-w PMC12227186

[13] Altaf A, Abbas MM. Public perception of bariatric surgery. Saudi Med J. 2019;40:378–83. 10.15537/smj.2019.4.24050 PMC6506652 30957132

[14] Lynch CS, Chang JC, Ford AF, et al. Obese African-American women’s perspectives on weight loss and bariatric surgery. J Gen Intern Med. 2007;22:908–14. 17447097 10.1007/s11606-007-0218-0 PMC2583799

[15] Slotman GJ. Gastric bypass: a family affair—41 families in which multiple members underwent bariatric surgery. Surg Obes Relat Dis. 2011;7:592–8. 21741322 10.1016/j.soard.2011.04.230

[16] Lent MR, Bailey-Davis L, Irving BA, et al. Bariatric surgery patients and their families: health, physical activity, and social support. Obes Surg. 2016;26:2981–8. 27173819 10.1007/s11695-016-2228-7 PMC5107175

[17] Husain F, Jeong IH, Spight D, et al. Risk factors for early postoperative complications after bariatric surgery. Ann Surg Treat Res. 2018;95:100–10. 30079327 10.4174/astr.2018.95.2.100 PMC6073041

[18] Hall RA. No assistance necessary: Arab men’s attitudes towards health issues and help-seeking. Open J Soc Sci. 2021;09:9–23.

[19] Fares S, Barajas-Gamboa JS, Díaz del Gobbo G, et al. Safety and efficacy of metabolic surgery in patients with type 2 diabetes in the Middle East and North Africa Region: an analysis of primary roux-en-Y gastric bypass and sleeve gastrectomy outcomes. J Clin Med. 2023;12:5077. 37568478 10.3390/jcm12155077 PMC10419696

[20] Hu Z, Sun J, Li R, et al. A comprehensive comparison of LRYGB and LSG in obese patients including the effects on QoL, comorbidities, weight loss, and complications: a systematic review and meta-analysis. Obes Surg. 2020;30:819–27. 31834563 10.1007/s11695-019-04306-4 PMC7347514

[21] Opozda M, Wittert G, Chur-Hansen A. Patients’ reasons for and against undergoing Roux-en-Y gastric bypass, adjustable gastric banding, and vertical sleeve gastrectomy. Surg Obes Relat Dis. 2017;13:1887–96. 28803707 10.1016/j.soard.2017.07.013

